# Awareness, Information-Seeking Behavior, and Information Preferences About Early Childhood Allergy Prevention Among Different Parent Groups: Protocol for a Mixed Methods Study

**DOI:** 10.2196/25474

**Published:** 2021-01-20

**Authors:** Jonas Lander, Janina Curbach, Julia von Sommoggy, Eva Maria Bitzer, Marie-Luise Dierks

**Affiliations:** 1 Hanover Medical School Hanover Germany; 2 University Hospital Regensburg Regensburg Germany; 3 Department of Public Health and Health Education Freiburg University of Education Freiburg Germany

**Keywords:** health literacy, allergy prevention, children, health information, parents

## Abstract

**Background:**

In early childhood allergy prevention (ECAP), parents act on behalf of their children. Parental health literacy and the availability of high-quality information, both online and offline, are crucial for effective ECAP. Recent research highlights three main points. First, parents need sufficient health literacy to discriminate between high-quality and low-quality information. Second, ECAP information behaviors may vary between phases of childhood development and according to individual circumstances. Third, to strengthen user-centeredness of available services, a better overview of parents’ information practices and needs and how they handle uncertainties is required.

**Objective:**

This study aims to explore why, how, and when parents search for and apply ECAP-specific health information and which individual (eg, understanding of advice) and organizational challenges (eg, information services, information complexity, and changing recommendations) they perceive and how they handle them. This study also aims to assess the needs and preferences that parents express for future information formats and contents. The findings should inform the practical design of ECAP information as well as formats and channels specific to different parent groups.

**Methods:**

The above-named issues will be explored with parents in four German cities as one element in our efforts to cover the spectrum of perspectives. Based on a mixed methods design, including qualitative and quantitative assessments, the first year serves to prepare focus groups, a piloted focus group guide, a short standardized survey adapted from the European Health Literacy Project, recruitment channels, and the recruitment of participants. After conducting 20 focus groups in the second year, data will be analyzed via a constant comparison method in the third year. Based on this, practice implications on channels (ie, Where?), formats (ie, How?), and contents (ie, What?) of ECAP-specific information will be derived and discussed with parents and associated project partners before its dissemination to relevant ECAP actors (eg, childcare institutions and pediatricians).

**Results:**

The study began with preselection of recruitment channels, drafting of recruitment and study information for potential participants, and agreement on a first full version of the guideline. Then, a detailed contact list was compiled of health professionals, administrative and social institutions, and relevant social media channels (N=386) to be approached for assistance in contacting parents. The recruitment was postponed due to COVID-19 and will start in January 2021.

**Conclusions:**

ECAP is a relevant example for assessing how users (ie, parents) handle not only health information but the various and continuous changes, uncertainties, and controversies attached to it. So far, it is unclear how parents implement the respective scientific recommendations and expert advice, which is why this study aims to inform those who communicate with parents about ECAP information.

**International Registered Report Identifier (IRRID):**

PRR1-10.2196/25474

## Introduction

### Background

#### Overview

The overall prevalence of children with allergies or asthma remains high and is often considered to be rising, despite the fact that exact numbers vary between regions [1-4]. While research on, and responses to, allergic diseases retain their traditional focus on treatment, nowadays prevention, immune system stimulation, and tolerance induction are considered increasingly important [5]. Hence, infants and young children may be a particularly important focus for prevention, because a person’s immune response to allergic triggers is formed early on in life [6]. For parents, this means that their role and the situation they find themselves in regarding early childhood allergy prevention (ECAP) has changed, particularly as new knowledge evolves continuously and often contradicts previous assumptions (eg, the effectiveness of deliberately exposing children to allergens, such as peanuts or eggs). This demands a more active, yet often less certain, parental role. Because of this and because recommendations given to parents change continuously [7-9], ECAP is a relevant example of how subject-specific health information is applied in daily (ie, regular) activities from four different perspectives, each with their own challenge.

#### Challenge 1: Information-Seeking Reasons and Motivations

Firstly, parents may search for information for different reasons and with varying motivations and individual circumstances [10]. Some may be concerned because of a known familial predisposition, some may have no particular risk but have more general concerns, while others may not have any specific interest. Apart from a particular risk status that triggers interest, different parental motivations may be evident at different stages of early childhood development. Also, a considerable proportion of parents may not seek ECAP-related information at all.

#### Challenge 2: Information Preferences

Secondly, evidence about parental information-seeking behavior and preferences regarding content, format, and delivery of information is crucial to adapt available services to parents’ specific requirements [11,12]. While there is ample evidence that many individuals lack health literacy—the ability to access, understand, appraise, and apply health information [13-15]—information regarding parents’ needs and preferences for ECAP-specific health information is scarce [16,17]. Research on needs and preferences relating to other topics suggests that available information is not tailored to target groups or according to the respective subjects [18]. It is also assumed that search behavior and preferences vary across different subjects and that parents are selective in seeking information (eg, during their children’s different developmental stages) [19].

#### Challenge 3: Information Formats

Thirdly, ECAP behavior and preferences may be influenced by available information formats. While the usage of online health information is increasing, the quality, safety, and reliability of available sources has been criticized in the past [20,21]. Further aspects, such as transparency, neutrality, appropriateness, and readability, may be equally important to ensure effectiveness [12,19,21,22]. This is also important because parents usually make use of more general information derived from public search engines rather than from specific sources for which a high degree of quality and a trustworthy evidence base can be assumed (ie, university- and health professional–authored sources) [12]. Another point of criticism of currently available online sources is that they are frequently overly technical, of limited accuracy, and contain too much information; in addition, there are no universal quality criteria for the development and provision of related services [18,23-28].

#### Challenge 4: Determinants of Information-Seeking Practices and Preferences

Lastly, parents’ information-seeking behavior and the respective stages and tasks related to searching, finding, appraising, and applying available information is influenced by their specific level of health literacy (see Methods section). For Germany, recent research highlights the prevalence of inadequate levels of health literacy in the population [13]. The association between health literacy and parents’ information and care practices has been discussed for other health issues (eg, obesity [29], food allergy [16], self-efficacy [30], weight control [31], and pediatric emergency utilization [32]). All studies highlight the negative effects of poor health literacy on parents’ health behavior; these studies suggest adjusting information sources and health care practices to health literacy levels [29] and developing strategies to support parents [30,33]. To develop respective materials and strategies, a better understanding of parents’ actual behavior and preferences is necessary [31]. In addition to the potential influence of health literacy, sociocultural habits, traditional beliefs, language barriers, past experiences, and routines regarding information use and access have been repeatedly described in the literature as important influencing factors. However, these aspects have not yet been assessed in the context of ECAP [34-37].

### Objectives

With the above-stated lack of empirical evidence on parental ECAP needs, preferences, and practices, the objectives of the planned study are shown in Table 1.

**Table 1:** Objectives and themes of the study.

Objective	Theme
Assess reasons and motivations for searching for early childhood allergy prevention (ECAP) information among different parent groups, and assess how these reasons are further influenced by parents’ awareness of, trust and uncertainty with, and beliefs of risks and myths associated with allergy prevention	Reasons, motivations, awareness, and trust
Explore how parents search for and apply ECAP information and clarify the emphasis on digital vis-à-vis nondigital sources	Information behavior
Describe preferences for information formats	Information formats (ie, needs and preferences)
Describe parents’ health literacy and sociocultural backgrounds and whether these create differences for the use of ECAP information	Influencing factors
Explore how sociocultural backgrounds may influence the above described aspects (ie, relevance, awareness, needs, preferences, and information behavior)	Sociocultural determinants
Summarize and disseminate key points for health professionals regarding parental handling of, and preferences for, ECAP information	Implications for practice

## Methods

### Study Design

#### Theory and Framework

The study of health literacy has gained considerable momentum, both at national (ie, Germany) and international levels (eg, [38-42]), due to the many people who have difficulties with accessing, understanding, appraising, and applying health information [15,43]. In light of its growing importance, the National Alliance for Health Literacy published the German National Action Plan for Health Literacy in 2018 [14]; similar initiatives are in place internationally, for instance, in Scotland and the United States [44,45]. Since the aim is to assess parental handling of, and needs regarding, ECAP, health literacy is at the core of each aspect of this study. The framework summarizes health literacy as a construct, with determinants of functional, interactive, and critical health literacy on a continuum from the individual to the population level, with the latter often being criticized for receiving too little attention [46,47].

**Figure 1:**
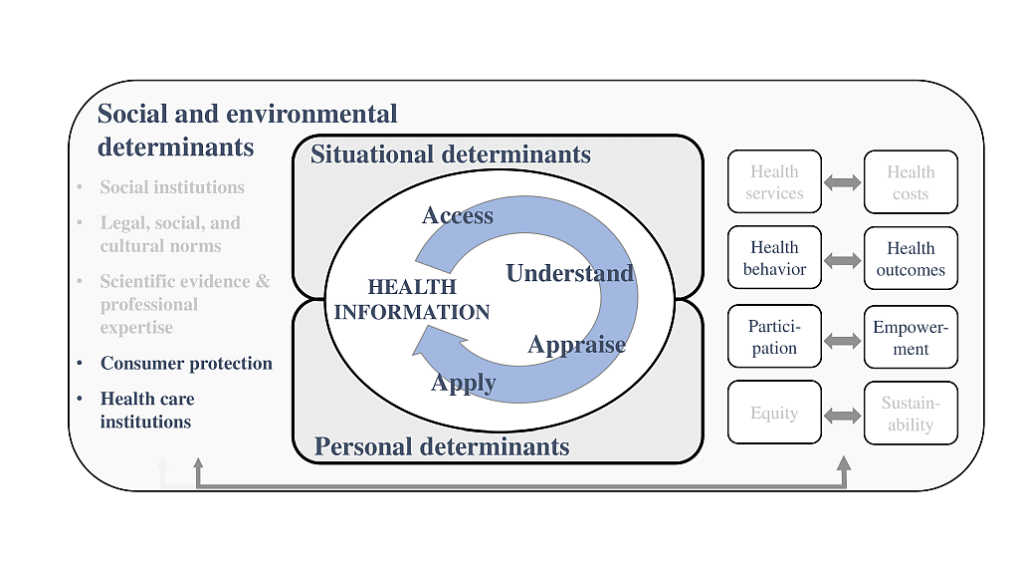
Health literacy framework.

The framework (see Figure 1) will be applied to (1) the development of the focus group manual, (2) data analysis (ie, development of coding categories; see Data Analysis section), and (3) formulation of recommendations, regarding both future ECAP information design and potential adjustments of the health literacy model. This framework has been slightly adapted from the original version by Sørensen et al [46] to emphasize the relevance of population-level determinants and the social embeddedness of health literacy.

#### Study Population

##### Target Group

To gather insights about the range of potentially different parental ECAP information behaviors and preferences and to help develop tailor-made future information materials and communication channels, different groups of parents will be recruited: (1) risk-specific groups (ie, parents with or without a specific risk of their child developing an allergy, based on the parents having or not having a medically confirmed allergy) and (2) life stage–specific groups (ie, expectant, new, and experienced parents).

Overall, a self-developed sampling matrix will be employed for a better overview of the characteristics of recruited participants and to identify parental characteristics that are underrepresented in the overall sample. The matrix will account for the above-mentioned main identifiers—allergy risk and life stage—as well as further, more typical recruitment criteria, including age, gender, educational status, migration background, and familial status (ie, living with or without a partner). The respective information will be queried during the recruitment process using a short questionnaire.

##### Recruitment Process

First, a list of contacts was compiled for health professionals (eg, pediatricians, gynecologists, and allergists) and for public and public health institutions (eg, kindergartens, family centers, and community offices) located in both urban and rural areas within a 10-km distance to the project sites in Germany (ie, Hanover, Magdeburg, Freiburg, and Regensburg) to reduce participants’ travel time. Respective contacts were searched for via (1) entries in medical online registers (ie, mainly online physician registries like Arztauskunft Niedersachsen), (2) coordinating bodies within public health (eg, the German Society for Allergy and Clinical Immunology, Landesamt für Gesundheit, and registries on insurance websites), and (3) general directories (eg, municipality websites, registries provided by municipalities for parents to find and choose a kindergarten or nursery, and local citizen administration and service offices). This will be complemented by (4) a Google search (eg, for further childcare institutions in specific cities) and (5) pre-established contacts (ie, mainly health professionals) from each project partner’s previous networks and collaborators of the project lead. From the overall list of contacts, those who can be expected to have the most direct and/or most frequent contact with the target group will be contacted first, such as kindergartens; we will contact further individuals and institutions if necessary. After completion of the search for recruitment agents, health professionals and institutions will be contacted personally to inquire about their support and availability for recruitment.

##### Recruitment Channels

As described above, parents will be approached via physician practices, public institutions, and a range of additional individual contacts from previously established project networks at the four project sites, in accordance with previous findings on the accessibility of lay target groups [48]. The first phase of recruitment is scheduled to take 1 to 2 months, followed by an assessment (ie, positive and negative recruitment channels) among the four project sites to identify difficulties. The second phase, expected to take 2 to 3 months, will then be conducted based on necessary adaptations.

Regarding recruitment via physician practices, access should be via (1) direct approach by the physician and/or nurses, for example, at the end of an appointment and (2) indirect approach via written information placed, for instance, at the clinic entrance or waiting room.

For recruitment via public institutions and offices, the main approach will be for an employee (eg, a nurse) to directly contact potential participants, for instance, for one hour in the morning, to limit the amount of staff time required. Again, an indirect approach via written information is also necessary to reach more potential participants. In settings where parents may best be reached at specific times of the day (eg, just before scheduled group meetings), project staff can support on-site recruitment. In addition, project sites’ institutions and institutions doing on-site recruitment will also use social media channels to post short messages with links to the project website. In advance, a search for relevant groups and websites was conducted, particularly private groups on Facebook.

Project staff have prepared written information used to inform potential participants about the project across the various recruitment channels. Feedback regarding its appropriateness, structure, and content has been gathered from the partner project on health professionals’ communication of ECAP information. Once participants have been selected, the initial short version of the project information document for participants used for recruitment will be adapted to an electronic version, using the online questionnaire tool SoSci Survey (SoSci Survey GmbH), to provide focus group participants with all necessary details. The electronic survey tool ensures full protection of personal data according to the German General Data Protection Regulation.

#### Data Collection

##### Qualitative Data Collection

A focus group approach seems valuable as it enables exchange among peers [49,50], with the subject of allergy-related prevention of health risks for the child being rather emotional and based on individual preferences, insights, and beliefs and, hence, worth discussing. In turn, this may help the target group reflect on their practices and needs based on others’ contributions and, hence, may stimulate additional input, particularly regarding potential adaptations of ECAP information formats and contents. A focus group manual was drafted and revised according to available research on the overarching subjects of health literacy, parental information behavior, and focus group methodology (see Multimedia Appendix 1) [12-14,16,34,51-54]. From these sources, individual themes were reframed as relevant questions for parents and ECAP. For example, the item “ability to judge the relevance of the information” translates into “When you read ECAP-specific online information, what contributes to your judgement of this information being relevant or not?” A pilot test with focus group participants (n=6) recruited locally in Hanover is scheduled 4 to 8 weeks in advance of the actual start of data collection.

**Figure 2:**
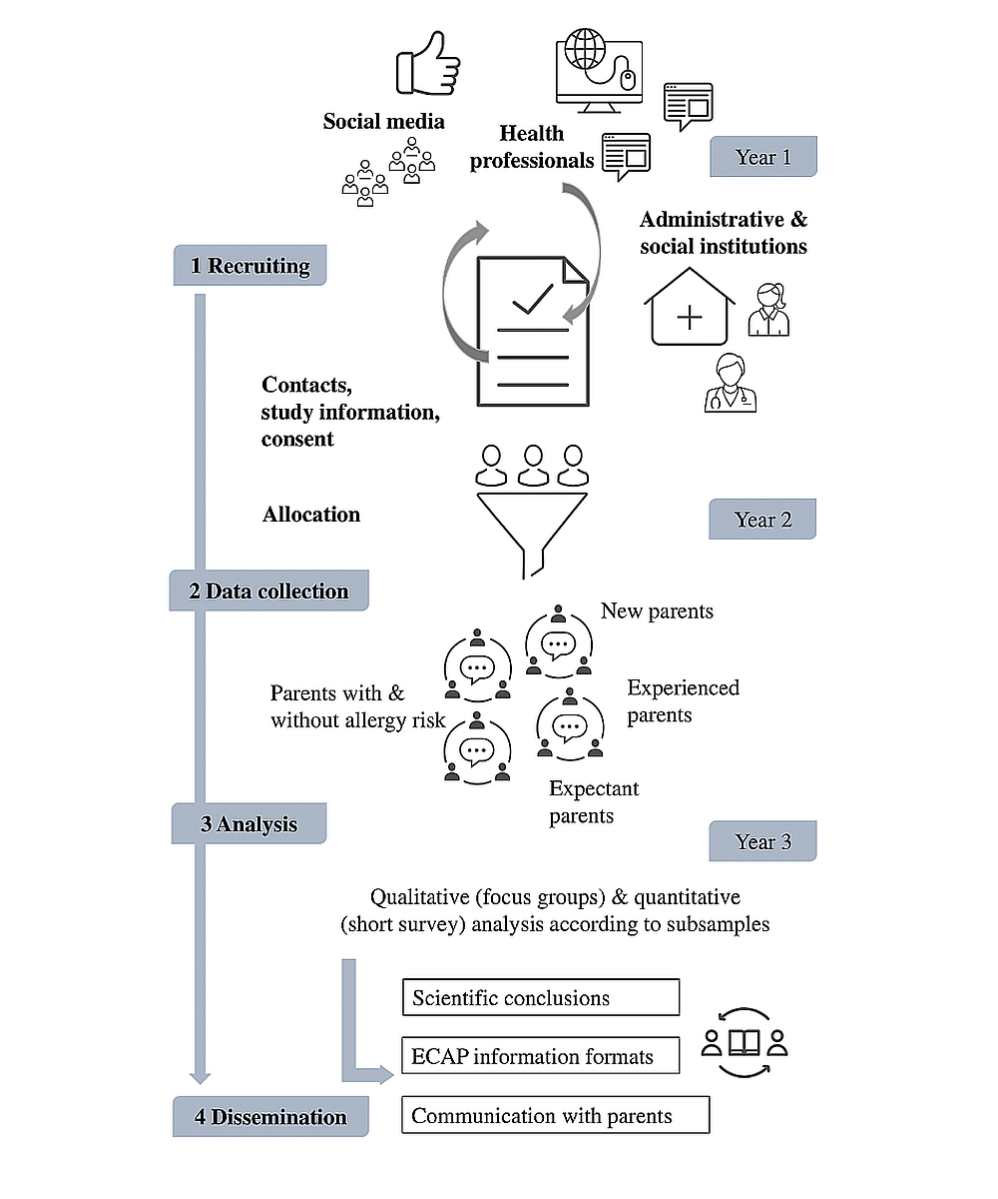
Main tasks and project phases. ECAP: early childhood allergy prevention.

Following the preparation phase, four to five focus groups will be conducted at each project site, structured around the risk-specific and life stage–specific parent groups (see Figure 2). Focus groups will be led by the project staff assisted by two more researchers to ensure good methodological practice. Each group will be scheduled for about 90 minutes with additional time to complete health literacy surveys (see Quantitative Data Collection section). After the initial conduct of four to five focus groups, potential adaptions regarding format, process, and content will be discussed and the remaining focus groups will be conducted accordingly.

##### Quantitative Data Collection

To assess and compare health literacy levels among the focus group participants (approximately N=120), a short version of the European Health Literacy Survey containing 16 items [52] will be applied, distributed, and completed alongside the focus group meetings. It covers three aspects of health along the four main health literacy dimensions, which appear suitable to assess health literacy in a group of parents. The survey comprises questions along the categories as shown in Table 2 as well as general information on each participant (eg, education status and health level), for a total of 25 questions.

**Table 2:** Subdimensions of health literacy, using the European Health Literacy Survey.

Health literacy subdimension	Information action			
	Access and obtain health information	Understand health information	Appraise and evaluate health information	Apply and use health information
Health care	Ability to access information on medical or clinical issues	Ability to understand medical information and derive meaning	Ability to interpret and evaluate medical information	Ability to make informed decisions on medical issues
Disease prevention	Ability to access information on risk factors	Ability to understand risk information and derive meaning	Ability to interpret and evaluate information on risk factors	Ability to judge the relevance of the information on risk factors
Health promotion	Ability to update oneself on health issues	Ability to understand health-related information and derive meaning	Ability to interpret and evaluate health-related information	Ability to form a considered opinion on health issues

#### Data Analysis

##### Parent Focus Groups

To account for potential differences in different parent groups (ie, risk-specific and life stage–specific groups), for instance, regarding when and how they seek ECAP information, the constant comparison method will be applied [55,56]. First, two focus groups will be selected from the risk-specific and life stage–specific parent groups, respectively, for detailed analysis and will be comprised of 6 participants each. Using established qualitative data analysis software, MAXQDA 2018 (Verbi GmbH), two researchers will independently apply open coding to attach codes (ie, descriptors will be applied to the various discussion sections). Codes will be broadly derived from the health literacy framework and focus group guideline items; additional codes will be added inductively as far as is relevant. Second, the various individual codes and respective text passages will be grouped into overarching categories. Next, the categories will be refined and structured around the initial research questions, again by two researchers, and core themes will be described for each. Any disagreement among the two researchers will be discussed with a third researcher, the project lead, to reach consensus. Each of the remaining focus groups will be analyzed using the set of previously built categories. Based on the final coding categories, key similarities and differences regarding information behavior, reasons for information searching, needs, and preferences, among others, will then be summarized and compared between groups.

##### Differences Based on Sociocultural Backgrounds

While the main part of the analysis will focus on the different parent groups, a separate round will be conducted to determine differences due to sociocultural backgrounds. To do so, the respective original transcripts will be screened for specific mentions (eg, preferences for a certain kind of information).

##### Health Literacy Survey

Data from the filled-in surveys will be entered into statistical data analysis software, SPSS Statistics, version 23 (IBM Deutschland GmbH), to allow for a descriptive portrayal of participant characteristics. This will be used mainly during the formulation of practice implications (see Expected Results section) (eg, specifying and characterizing parent groups that demand specific information formats and contents [ie, “Who needs what?”]). Individual health literacy levels will be indicated as percentage shares for items rated as *very easy*, *rather easy*, *rather difficult*, *very difficult*, and *don’t know*. Individual ratings will then be displayed as *excellent*, *adequate*, *insufficient*, and *problematic* according to the differentiation of health literacy levels [15]. Also, health literacy levels will be compared among the different parent groups based on mean values.

#### Next Steps for Recruitment and Focus Group Discussion Manual

As of January 2020, the study started with a structured online search for (1) clinics, (2) general practitioners, (3) gynecologists, (4) pediatricians, (5) allergists, (6) midwives, (7) kindergartens, (8) public administration offices, and (9) local social institutions across the different local project sites, according to the plan described in the Recruitment Process section, to use these individuals and institutions for direct access to the target groups. Broad inclusion criteria were defined in cases where there were too many potential contacts. For example, general practitioners were included if they had a professional website, provided an email address, operated as a joint practice with at least two physicians, and had a medical degree. The contacts were further limited to a maximum of 15 per category to keep the subsequent contacting manageable. The current list entails 386 contacts across all project sites; further contacts may be added when necessary (eg, family centers and community offices with specific services for migrant parents).

Then, a manual for the focus group discussions was drafted, based on a search in PubMed and Google Scholar for available research on focus groups with parents on the subject of prevention measures for children (eg, [57-59]) and on established and alternative methods for the structure and conduct of focus groups [60-62]. All relevant sources were screened for input on methods and content of the planned focus groups. Summaries of each finding were implemented in the initial draft version of our manual following discussions among the project staff. A first complete version of the manual has been agreed on internally; further feedback will be gathered within the research unit once recruitment is underway. The manual shall also be sent to a small group of health professionals for content-related feedback (eg, an allergist from the allergy clinic of our host institution; a health literacy expert, to be identified from the scientific advisory board established as part of the research unit; and a local public health or health care institution, such as the German Allergy and Asthma Foundation).

#### Transferring Results to Practice

##### Step 1: Participatory Development of Practice Implications

Based on our findings, our aim is to determine (1) where ECAP-specific information should be placed to reach more users and what motivates parents to consider information, (2) the topics that create uncertainties (eg, recommendations on allergenic foods) and what parents consider helpful for navigating such challenges, and (3) the preferred information and learning formats and respective variations across parent groups.

To derive practice implications, relevant codes from the analysis will be grouped under each aspect. A preliminary set of implications will be drafted according to the categorization of codes, for which (1) volunteers from the focus groups (n=5) as well as (2) a group of health professionals (n=5) shall be invited to comment on, revise, and consent to the practice implications. The latter will be approached based on one of our neighboring projects within the Health Literacy in Early Childhood Allergy Prevention (HELICAP) research unit, which interviews health professionals from different disciplines regarding their ECAP communication with parents. A final version will be created based on the overall feedback; the exact format (eg, a user-friendly visualized brochure) will be consented to during the development process.

##### Step 2: Dissemination

Practice implications will primarily be disseminated to health professionals who inform and consult parents about ECAP (ie, midwives, pediatricians, and allergists) and providers of digital health information. Also, childcare service institutions, such as kindergartens and family centers, will be invited to offer short summaries of the main findings from both provider and user perspectives within their institution. Formats other than written summaries might be necessary here, which still must be agreed upon in the course of the project. To approach the above-mentioned individual actors, a combination of dissemination channels will be employed, particularly the following:

1. All actors and institutions for which contact details were collected for the recruitment of parents.
2. Established collaborations and networks by key German actors in the field of allergy-specific health information (eg, the German Allergy Information Service and the German Allergy and Asthma Foundation).
3. Umbrella organizations that reach out to allergy experts (eg, the German Society for Pediatric Allergology) and family and childcare institutions (eg, the Federal Association of Family Centers).

##### Step 3: Preparing the Findings for Intervention Development and Testing in a Second Project Phase

A final step is to transfer the study results and practice implications into a subsequent phase of intervention development and testing. At this point, a set of ECAP-specific information materials would be drafted based on the project described here as well as on the recommendations for evidence-based health information (eg, [63-65]). This could then be piloted via a randomized controlled trial (ie, one group of parents receiving the intervention and another group receiving currently available ECAP information). A specific focus for the information materials (eg, suggestions for dealing with uncertainty) will be specified if the results of this study demonstrate a need for it. Besides constant patient and public involvement during the development stage by a group of parents, the development phase would be further supported by allergy prevention experts. These experts would be approached, for instance, via the associated university hospitals and respective allergy and pediatric clinics at each project site.

#### Ethical Considerations

Besides a deeper reflection on the participants’ own ECAP prevention and information practices and those of other focus group participants, as well as potentially controversial discussions, there are no likely risks to be expected from the conduct of focus groups. The study design, including the recruitment, conduct, and analysis, has been approved by the Ethics Committee of Hanover Medical School (ID 8161_BO_K_2018).

## Results

The study began with preselection of recruitment channels, drafting of recruitment and study information for potential participants, and agreement on a first full version of the guideline. Then, a detailed contact list was compiled of health professionals, administrative and social institutions, and relevant social media channels (N=386) to be approached for assistance in contacting parents. Given the COVID-19 pandemic and, hence, the substantially limited access to potential participants as well as restrictions for direct meetings, it was decided, in accordance with the funding agency, to pause the recruitment and restart this process as of January 2021. Presumably, focus groups will commence in the first quarter of 2021, and an alternative to direct meetings is being considered in the case of continuing contact restrictions.

## Discussion

A major step toward successful focus group conduct is the recruitment of a sample diverse in individual backgrounds and perspectives, for example, by including parents with and without allergic predispositions, expectant and more experienced parents, single parents, and those who are not native German speakers. Hence, it will be important to sensitize recruitment agents to this issue, to contact institutions supporting disadvantaged people, and to ask all participants to inform peers.

The inclusion of hard-to-reach groups (ie, those that do not receive the call for study participation, those who do not use the internet, those who do not have the time or resources for participation, those who feel uncomfortable participating in group discussions, those with low reading levels, and those who have difficulties reading German) could prove difficult. While this potential challenge cannot necessarily be circumvented completely, we will make sure to address individual actors and organizations with specific access to these groups, particularly family and childcare organizations with specific services (ie, *Familienhilfe*) and family centers located in social flashpoints. Also, recruitment could be done directly from low-education suburbs (eg, via distributing study flyers directly to each household). It will also be important to inform, for example, family centers’ staff early, discuss potentially necessary adaptations of the recruitment material (eg, language), and ask them to approach potential participants more directly (ie, in their role as a reference person for hard-to-reach groups).

Experiences from similar, yet unpublished, studies on parental health literacy conducted by the research team in Hanover will provide valuable help for structuring the discussions on ECAP, addressing issues that deserve particular attention (eg, reasons for not adhering to certain sources and revising or changing less relevant topics). The challenge here may be to go beyond a discussion of general information behaviors (eg, Googling) and trusted sources (eg, scientific experts), though not neglecting these issues, and instead emphasize issues such as trust, decision making, and health literacy skill development.

As the COVID-19 pandemic continues to impede social contacts on-site, an alternative to this study’s planned focus group methodology will have to be considered, particularly online (ie, video) meeting tools [62]. While the organizational and technical aspects (eg, unrestricted access to the tool, sending log-in details, providing usage information, and ensuring proper functioning) may all be implemented in advance, it is unclear if an online meeting will be suitable, particularly regarding whether participants would contribute the same information as they would in a face-to-face meeting. For instance, it may be more difficult to react to another person’s statement when not being able to observe the entire group and reactions by others. This would, in turn, impede group dynamics and result in a more passive discussion. However, reducing the size of participants per online focus group (eg, maximum of 5), thereby conducting more online focus groups overall, may contribute to improved perceptions of belonging and, hence, to participants contributing with greater confidence.

To conclude, with this study, the understanding of parents’ information behavior and needs with respect to ECAP shall be improved, as allergies (ie, atopic diseases) are a major health issue in western industrialized societies that demand timely prevention strategies. It is hoped that we may not only develop a deeper understanding of individual influences regarding a person’s ability to handle health information but also that we may gain insight into external organizational factors shaping individual health literacy, which have been largely neglected by previous attempts to create a comprehensive understanding of health literacy. The findings should also help generate practical advice for health professionals, public institutions, and public health institutions on providing user-centered information materials as well as for parents to access information resources that help them (1) to deal with uncertainty and risk, (2) not to be misled by inaccurate sources, and (3) to make informed choices about child health.

## Appendix

Multimedia Appendix 1 Focus group guideline.

## Abbreviations

ECAP: early childhood allergy prevention
HELICAP: Health Literacy in Early Childhood Allergy Prevention

## References

[1] Pawankar R (2014). Allergic diseases and asthma: A global public health concern and a call to action. World Allergy Organ J.

[2] (2018). Global Strategy for Asthma Management and Prevention.

[3] Croisant S (2014). Epidemiology of asthma: Prevalence and burden of disease. Adv Exp Med Biol.

[4] Simon D (2018). Recent advances in clinical allergy and immunology. Int Arch Allergy Immunol.

[5] Fisher HR, Du Toit G, Bahnson HT, Lack G (2018). The challenges of preventing food allergy: Lessons learned from LEAP and EAT. Ann Allergy Asthma Immunol.

[6] Du Toit G, Roberts G, Sayre PH, Bahnson HT, Radulovic S, Santos AF, Brough HA, Phippard D, Basting M, Feeney M, Turcanu V, Sever ML, Gomez Lorenzo M, Plaut M, Lack G (2015). Randomized trial of peanut consumption in infants at risk for peanut allergy. N Engl J Med.

[7] Genuneit J, Seibold AM, Apfelbacher CJ, Konstantinou GN, Koplin JJ, La Grutta S, Logan K, Perkin MR, Flohr C, Task Force ‘Overview of Systematic Reviews in Allergy Epidemiology (OSRAE)’ of the EAACI Interest Group on Epidemiology (2017). Overview of systematic reviews in allergy epidemiology. Allergy.

[8] Perkin MR, Logan K, Marrs T, Radulovic S, Craven J, Flohr C, Lack G, EAT Study Team (2016). Enquiring About Tolerance (EAT) study: Feasibility of an early allergenic food introduction regimen. J Allergy Clin Immunol.

[9] Perkin MR, Logan K, Tseng A, Raji B, Ayis S, Peacock J, Brough H, Marrs T, Radulovic S, Craven J, Flohr C, Lack G, EAT Study Team (2016). Randomized trial of introduction of allergenic foods in breast-fed infants. N Engl J Med.

[10] Hu W, Grbich C, Kemp A (2008). When doctors disagree: A qualitative study of doctors' and parents' views on the risks of childhood food allergy. Health Expect.

[11] Harmsen IA, Doorman GG, Mollema L, Ruiter RAC, Kok G, de Melker HE (2013). Parental information-seeking behaviour in childhood vaccinations. BMC Public Health.

[12] Pehora C, Gajaria N, Stoute M, Fracassa S, Serebale-O'Sullivan R, Matava CT (2015). Are parents getting it right? A survey of parents' internet use for children's health care information. Interact J Med Res.

[13] Schaeffer D, Berens E, Vogt D (2017). Health literacy in the German population. Dtsch Arztebl Int.

[14] Schaeffer D, Hurrelmann K, Bauer U, Kolpatzik K (2018). National Action Plan Health Literacy. Promoting Health Literacy in Germany [Document in German].

[15] Sørensen K, Pelikan JM, Röthlin F, Ganahl K, Slonska Z, Doyle G, Fullam J, Kondilis B, Agrafiotis D, Uiters E, Falcon M, Mensing M, Tchamov K, van den Broucke S, Brand H, HLS-EU Consortium (2015). Health literacy in Europe: Comparative results of the European Health Literacy Survey (HLS-EU). Eur J Public Health.

[16] Ditzler N, Greenhawt M (2016). Influence of health literacy and trust in online information on food allergy quality of life and self-efficacy. Ann Allergy Asthma Immunol.

[17] Kayser L, Kushniruk A, Osborne RH, Norgaard O, Turner P (2015). Enhancing the effectiveness of consumer-focused health information technology systems through eHealth literacy: A framework for understanding users' needs. JMIR Hum Factors.

[18] Lee K, Hoti K, Hughes JD, Emmerton L (2014). Dr Google and the consumer: A qualitative study exploring the navigational needs and online health information-seeking behaviors of consumers with chronic health conditions. J Med Internet Res.

[19] Walsh AM, Hamilton K, White KM, Hyde MK (2015). Use of online health information to manage children's health care: A prospective study investigating parental decisions. BMC Health Serv Res.

[20] Eysenbach G, Powell J, Kuss O, Sa E (2002). Empirical studies assessing the quality of health information for consumers on the world wide web: A systematic review. JAMA.

[21] Lander J, Drixler K, Dierks M, Bitzer EM (2019). How do publicly available allergy-specific web-based training programs conform to the established criteria for the reporting, methods, and content of evidence-based (digital) health information and education: Thematic content evaluation. Interact J Med Res.

[22] American Public Health Association (2001). Criteria for assessing the quality of health information on the internet. Am J Public Health.

[23] Silver MP (2015). Patient perspectives on online health information and communication with doctors: A qualitative study of patients 50 years old and over. J Med Internet Res.

[24] Sillence E, Briggs P, Harris PR, Fishwick L (2007). How do patients evaluate and make use of online health information?. Soc Sci Med.

[25] Stvilia B, Mon L, Yi YJ (2009). A model for online consumer health information quality. J Am Soc Inf Sci Technol.

[26] Beaunoyer E, Arsenault M, Lomanowska AM, Guitton MJ (2017). Understanding online health information: Evaluation, tools, and strategies. Patient Educ Couns.

[27] Cherla DV, Sanghvi S, Choudhry OJ, Liu JK, Eloy JA (2012). Readability assessment of internet-based patient education materials related to endoscopic sinus surgery. Laryngoscope.

[28] Diviani N, van den Putte B, Meppelink CS, van Weert JCM (2016). Exploring the role of health literacy in the evaluation of online health information: Insights from a mixed-methods study. Patient Educ Couns.

[29] Cha E, Besse JL (2015). Low parent health literacy is associated with 'obesogenic' infant care behaviours. Evid Based Nurs.

[30] Fong H, Rothman EF, Garner A, Ghazarian SR, Morley DS, Singerman A, Bair-Merritt MH (2018). Association between health literacy and parental self-efficacy among parents of newborn children. J Pediatr.

[31] Liechty JM, Saltzman JA, Musaad SM, STRONG Kids Team (2015). Health literacy and parent attitudes about weight control for children. Appetite.

[32] Morrison AK, Myrvik MP, Brousseau DC, Hoffmann RG, Stanley RM (2013). The relationship between parent health literacy and pediatric emergency department utilization: A systematic review. Acad Pediatr.

[33] Jimenez ME, Barg FK, Guevara JP, Gerdes M, Fiks AG (2013). The impact of parental health literacy on the early intervention referral process. J Health Care Poor Underserved.

[34] King-Shier K, Lau A, Fung S, LeBlanc P, Johal S (2018). Ethnocultural influences in how people prefer to obtain and receive health information. J Clin Nurs.

[35] Lee SK, Sulaiman-Hill CMR, Thompson SC (2013). Providing health information for culturally and linguistically diverse women: Priorities and preferences of new migrants and refugees. Health Promot J Austr.

[36] Walker LO, Mackert MS, Ahn J, Vaughan MW, Sterling BS, Guy S, Hendrickson S (2017). eHealth and new moms: Contextual factors associated with sources of health information. Public Health Nurs.

[37] Singh P, Hayden KA, Ens T, Khan N, Quan H, Plested D, Sinclair S, King-Shier KM (2017). Ethno-cultural preferences in receipt of heart health information. Am J Health Behav.

[38] Bröder J, Okan O, Bauer U, Bruland D, Schlupp S, Bollweg TM, Saboga-Nunes L, Bond E, Sørensen K, Bitzer E, Jordan S, Domanska O, Firnges C, Carvalho GS, Bittlingmayer UH, Levin-Zamir D, Pelikan J, Sahrai D, Lenz A, Wahl P, Thomas M, Kessl F, Pinheiro P (2017). Health literacy in childhood and youth: A systematic review of definitions and models. BMC Public Health.

[39] Chen X, Hay JL, Waters EA, Kiviniemi MT, Biddle C, Schofield E, Li Y, Kaphingst K, Orom H (2018). Health literacy and use and trust in health information. J Health Commun.

[40] Abel T, Sommerhalder K (2015). Health literacy: An introduction to the concept and its measurement [Article in German]. Bundesgesundheitsblatt Gesundheitsforschung Gesundheitsschutz.

[41] Smith SK, Nutbeam D, McCaffery KJ (2013). Insights into the concept and measurement of health literacy from a study of shared decision-making in a low literacy population. J Health Psychol.

[42] Batterham RW, Beauchamp A, Osborne RH, Quah SR, Cockerham WC (2017). Health literacy. International Encyclopedia of Public Health. 2nd edition.

[43] Berens E, Vogt D, Ganahl K, Weishaar H, Pelikan J, Schaeffer D (2018). Health literacy and health service use in Germany. Health Lit Res Pract.

[44] NHS Scotland (2017). Making it Easier: A Health Literacy Action Plan for Scotland 2017-2025.

[45] (2010). National Action Plan to Improve Health Literacy.

[46] Sørensen K, Van den Broucke S, Fullam J, Doyle G, Pelikan J, Slonska Z, Brand H, (HLS-EU) Consortium Health Literacy Project European (2012). Health literacy and public health: A systematic review and integration of definitions and models. BMC Public Health.

[47] Farmanova E, Bonneville L, Bouchard L (2018). Organizational health literacy: Review of theories, frameworks, guides, and implementation issues. Inquiry.

[48] Drixler K, Lander J, Luntz E, Schäfer I, Schmitt J, Dierks ML, Bitzer EM (2018). Projektzwischenbericht PAKO-ATOP für den Berichtszeitraum 01.04.2017-30.04.2018.

[49] Côté-Arsenault D, Morrison-Beedy D (2005). Maintaining your focus in focus groups: Avoiding common mistakes. Res Nurs Health.

[50] Tausch A, Menold N (2015). Methodische Aspekte der Durchführung von Fokusgruppen in der Gesundheitsforschung - Welche Anforderungen ergeben sich aufgrund der besonderen Zielgruppen und Fragestellungen? [Methodological Aspects of Conducting Focus Groups in Health Research - Which Requirements Result From the Particular Target Groups and Research Questions?]. GESIS Papers.

[51] Schulz M, Mack B, Renn O (2012). Fokusgruppe in der empirischen Sozialwissenschaft: Von der Konzeption bis zur Auswertung.

[52] The HLS-EU Consortium (2012). Measurement of health literacy in Europe: HLS-EU-Q47; HLS-EU-Q16; and HLS-EU-Q86. The European Health Literacy Project 2009-2012.

[53] Hu W, Grbich C, Kemp A (2007). Parental food allergy information needs: A qualitative study. Arch Dis Child.

[54] Lovell JL (2016). How parents process child health and nutrition information: A grounded theory model. Appetite.

[55] Corbin J, Strauss A (2015). Basics of Qualitative Research: Techniques and Procedures for Developing Grounded Theory. 4th edition.

[56] Denzin NK, Lincoln YS (2017). The SAGE Handbook of Qualitative Research. 5th edition.

[57] De Lepeleere S, DeSmet A, Verloigne M, Cardon G, De Bourdeaudhuij I (2013). What practices do parents perceive as effective or ineffective in promoting a healthy diet, physical activity, and less sitting in children: Parent focus groups. BMC Public Health.

[58] Harmsen IA, Bos H, Ruiter RAC, Paulussen TGW, Kok G, de Melker HE, Mollema L (2015). Vaccination decision-making of immigrant parents in the Netherlands: A focus group study. BMC Public Health.

[59] Gupta RS, Kim JS, Barnathan JA, Amsden LB, Tummala LS, Holl JL (2008). Food allergy knowledge, attitudes and beliefs: Focus groups of parents, physicians and the general public. BMC Pediatr.

[60] Quach S, Pereira JA, Russell ML, Wormsbecker AE, Ramsay H, Crowe L, Quan SD, Kwong J (2013). The good, bad, and ugly of online recruitment of parents for health-related focus groups: Lessons learned. J Med Internet Res.

[61] Cobb G (2017). Focus groups: Creativity killers. Ideas To Go.

[62] Tates K, Zwaanswijk M, Otten R, van Dulmen S, Hoogerbrugge PM, Kamps WA, Bensing JM (2009). Online focus groups as a tool to collect data in hard-to-include populations: Examples from paediatric oncology. BMC Med Res Methodol.

[63] Charnock D, Shepperd S (2004). Learning to DISCERN online: Applying an appraisal tool to health websites in a workshop setting. Health Educ Res.

[64] On the Net (2000). HON code of conduct for medical and health web sites. Am J Health Syst Pharm.

[65] Winker MA, Flanagin A, Chi-Lum B, White J, Andrews K, Kennett RL, DeAngelis CD, Musacchio RA (2000). Guidelines for medical and health information sites on the internet: Principles governing AMA web sites. JAMA.

[66] HELICAP Research Group (2020). Health Literacy in Early Childhood Allergy Prevention. HELICAP.

