# Correction: Awareness, Information-Seeking Behavior, and Information Preferences About Early Childhood Allergy Prevention Among Different Parent Groups: Protocol for a Mixed Methods Study

**DOI:** 10.2196/42011

**Published:** 2022-09-09

**Authors:** Jonas Lander, Janina Curbach, Julia von Sommoggy, Eva Maria Bitzer, Marie-Luise Dierks

**Affiliations:** 1 Hanover Medical School Hanover Germany; 2 University Hospital Regensburg Regensburg Germany; 3 Department of Public Health and Health Education Freiburg University of Education Freiburg Germany

In “Awareness, Information-Seeking Behavior, and Information Preferences About Early Childhood Allergy Prevention Among Different Parent Groups: Protocol for a Mixed Methods Study” (JMIR Res Protoc 2021;10(1):e25474), the authors noted one error.

1. In the originally published article, the *Acknowledgments* section inadvertently appeared with an incomplete funding statement as follows:

*We would like to thank Reuben Thomas very much for detailed language support. We would also like to thank the initial members of the research group HELICAP [​66​], funded by the German Research Foundation, of which this project is a part: Christian Apfelbacher, Eva Maria Bitzer, Uwe Matterne, Nina Egger, Janina Curbach, Julia von Sommoggy, Susanne Brandstetter, Maja Pawellek, Carolin Dresch, Anja Schulz, and Markus Wirtz. We would also like to thank the external grant proposal advisors—Julika Loss and Kristine Sørensen—and the grant proposal reviewers who all provided valuable and critical feedback on the conceptualization of the study, its methodological design, and its integration into the overarching goals and structure of the research group.*

In the corrected version, the *Acknowledgments* section has been updated as follows:

We would like to thank the steering committee (Christian Apfelbacher, Eva Maria Bitzer, Janina Curbach, Marie-Luise Dierks, Susanne Brandstetter, Markus Antonius Wirtz) as well as the initial members (Nina Egger, Christina Tischer, Jana Tempes, Uwe Matterne, Julia von Sommoggy, Maja Pawellek, Carolin Dresch, Anja Schulz) of the research group “Health Literacy in Early Childhood Allergy Prevention” (HELICAP, [​66​]). HELICAP is funded by the German Research Foundation (DFG FOR 2959, project-id: 409800133), of which this project is a part (grant number: DI 1757/2-1). The funder had no role in the design of the study, data collection, data analysis, or interpretation of the results. We also thank the external grant proposal advisers (Julika Loss, Kristine Sørensen) and the grant proposal reviewers who all provided valuable and critical feedback on the conceptualization of the study, its methodological design, and its integration into the overarching goals and structure of the research group. Lastly, we are grateful for the detailed language editing support provided by Roy Reuben Thomas.

The correction will appear in the online version of the paper on the JMIR Publications website on September 9, 2022, together with the publication of this correction notice. Because this was made after submission to PubMed, PubMed Central, and other full-text repositories, the corrected article has also been resubmitted to those repositories.

