# Supplementary material for: Awareness, Information-Seeking Behavior, and Information Preferences About Early Childhood Allergy Prevention Among Different Parent Groups: Protocol for a Mixed Methods Study
Source: JMIR Res Protoc. 2021 Jan 20;10(1):e25474. doi: 10.2196/25474 (PMC7857939; doi:10.2196/25474)
Supplement: Multimedia Appendix 1 [file resprot_v10i1e25474_app1.pdf]

Focus group guideline - USER NEEDS

1. Organization

follows

2. Positioning

Answer 3 questions on the flipchart with adhesive dots

(agree completely - do not agree at all)

- I am very interested in the topic "allergies and children
- I find the Internet very helpful when it comes to "allergies in children
- I am afraid that I might make the wrong decisions about how to deal with allergies in my child

3. Welcome (5)

- Greetings and thanks
- Presentation moderators
- Background and objectives of the study
- Conversation process (topics, time, 'rules')
- Explanations: Tape recording, questionnaire, data storage, consent, reimbursement

4. Participants introduction (20)

- I am... and I'm from...
- I am interested in the topic of allergies in children because... / When I think of the topic "allergies in children", then I think of the fact that... / on the topic of allergies in children I have recently heard that...
- Inquiries based on participants information

5. Topic awareness, knowledge, relevance (25)

Imagine that you have read in the Allergy Information Service that almost one in four children and adolescents suffer from an allergy.

- What do you think about this information?
- Does the risk of a possible allergy or the already existing allergy in your child worry you in any way? Why, why not?
- Is the topic even important to you?
- Why, why not?

What do you yourself know about allergies in children, what have you heard about them?

- Do you know of any specific recommendations for dealing with allergies in children?
- ▪ Are the recommendations helpful for you, do you follow them?
- Have you ever had the impression that information is contradictory or wrong?
- How did you deal with this?
- Have you ever thought about the quality of information, e.g. whether an information is scientifically proven?

- Does this influence your search / handling of information?

Note: here only very short information, no long stories

6. Information behavior, challenges (25)

Scenario a - parents with risk and without risk Imagine you read on a well-known site on the Internet that excessive washing, cleaning, disinfecting and avoiding pets increases the risk of allergy in your child. But you yourself always have a very clean home and pay attention to hygiene. Now they are not sure if they should change anything when you have a child / have a child.

Scenario b - Becoming parents and new parents Imagine you are at the pediatrician or midwife. You talk about the topic of nutrition and learn that it makes no sense to avoid certain foods in babies and toddlers and that even children with an increased risk of allergies should not do without anything. After the conversation you want to read more about this on the Internet and read in a parents' forum that many parents for example avoid cow's milk, eggs, wheat, carrots, nuts (from the 5th month). Now you are no longer so sure whether the information from the doctor is really correct.

Scenario c - Experienced parents Imagine you are at the pediatrician with your daughter or son (with allergies). You talk about the topic of allergy treatment and learn that an allergy vaccination (hyposensitization) is quite possible from the age of 6 years. At home you want to inform yourself further about this. In a forum many parents report that the therapy takes far too long, is expensive and in the end does not help anything. Now you are no longer so sure whether the information from the doctor is really correct and whether the treatment would be good for your child.

- What would be important for you to get clarity in this situation?
- Where would you look for information and how?
- When you think of the Internet,
  - Which sources are helpful for you?
  - How do you decide what to choose in Google?
  - What about social media, is it relevant to you when it comes to allergies in children?
- If you were to talk to the doctor about your information from the Internet again afterwards, how could the doctor help you?
- What about friends, family?
  - Do you talk to them about the topic?
  - What do you do with the information?

- Do you generally make a difference between information from experts and laymen?

If you think again about weighing up information (as in the example)

- Are you often or rarely uncertain when it comes to making decisions about your child's health? Why?
- What is the reason for the uncertainty?
- What would help to make you less insecure?

7. Needs, future design (40)

- The participants will give to divide themselves into 3 groups, each group will receive a link to the topic "Prevent allergies".
- Work order (time: 10 min)

Please look at the page and discuss together

- How helpful is the information?
- Are they serious, why?
- What is good about it, what is not good? Why?
- What do you miss?

>> the participants receive an A4 page with given questions

Group 1 - Working group allergy ill child: Avoidance of irritants and allergens in the house https://www.aak.de/allergie-bei-kindern/schutz-vor-allergien/vermeidung-von-reizstoffen-und-allergenen-im-haeuslichen-bereich/

Group 2 - Allergy information service: Preventing allergies in children https://www.allergieinformationsdienst.de/vorbeugung-schutz/kinder-und-allergien/vorbeugung.html

Group 3 - Star online: The big snort https://www.stern.de/gesundheit/allergie/erkrankungen/allergie-bei-kindern-das-grosse-schnaufen-3360886.html

- Afterwards: each group briefly presents its results (5 min per group)

Subsequent questions

- If you now look at the results from the 3 groups, what makes an information a good information?
- What do you consider to be the most important questions about allergies in children?
- What kind of information do you find good and why?
    - Text, video, forum, chat, ...
    - From whom should the information important for you come?
    - What helps you to make decisions, e.g. whether you should avoid a certain food for your child?

8. Wrap up, questions, further procedure, goodbye

Based on respective group and comments
